# Clinical and Cellular Evidence of *Glycyrrhiza glabra* and *Platycodon grandiflorus* for Vocal Fold Nodules Complementary Treatment

**DOI:** 10.7150/ijms.68118

**Published:** 2022-05-01

**Authors:** I-Yun Lee, Shun-Fu Chang, Chin-Yuan Wu, Yu-Shi Lin, Hui-Chen Su, Yao-Te Tsai, Ming-Shao Tsai, Geng-He Chang, Ming-Yu Yang, Yao-Hsu Yang, Pei-Rung Yang, Cheng-Ming Hsu

**Affiliations:** 1 Department of Chinese Medicine, Chiayi Chang Gung Memorial Hospital, Chiayi 61363, Taiwan.; 2 School of Chinese Medicine, College of Medicine, Chang Gung University, Taoyuan 33302, Taiwan.; 3 Department of Medical Research and Development, Chiayi Chang Gung Memorial Hospital, Chiayi 61363, Taiwan.; 4 Department of Pharmacy, Chiayi Chang Gung Memorial Hospital, Chiayi, Taiwan.; 5 Department of Neurology, National Cheng-Kung University Hospital, Tainan, Taiwan.; 6 Department of Otolaryngology-Head and Neck Surgery, Chiayi Chang Gung Memorial Hospital, Chiayi 61363, Taiwan.; 7 Graduate Institute of Clinical Medical Sciences, College of Medicine, Chang Gung University, Taoyuan 33302, Taiwan.; 8 Department of Otolaryngology, Kaohsiung Chang Gung Memorial Hospital, Kaohsiung 83301, Taiwan.; 9 School of Medicine, College of Medicine, Chang Gung University, Taoyuan 33302, Taiwan.

**Keywords:** vocal fold nodules, traditional Chinese medicine, herbs, *Glycyrrhiza glabra*,* Platycodon grandifloras*

## Abstract

Vocal fold nodules (VFNs) are the most frequent cause of hoarseness. The management comprised medical, surgical and physical therapy but the effectiveness is not always satisfactory. In this study, we try to figure out an alternative treatment from our clinical experience summary. We retrospectively reviewed VFNs patients who received traditional Chinese medicine (TCM) treatments from July 2018 to August 2020 and traced their Chinese Voice Handicap Index-10 (VHI-C10) and multidimensional voice program (MDVP) analysis results. For further evaluation, we conducted an inflammatory response of porcine vocal fold epithelial (PVFE) cells with 50 ng/mL TNF-alpha. The inflamed PVFE cells were separately cultured in the aqueous extract of Glycyrrhiza glabra (*G. glabra*) and Platycodon grandifloras (*P. grandifloras*). In these VFNs patients (n = 22), the average VHI-C10 score decreased from 17.6 to 6.6 (p < 0.001). MDVP analysis revealed improvements in jitter, shimmer, noise-harmonic ratio, and GRBAS scoring system. Of the TCM prescription patterns, *G. glabra* and *P. grandiflorus* were used most frequently. In the MTT assay of PVFE cells, no adverse effects of our extracts were observed at doses of 1-200 µg/mL. Western blot analysis revealed downregulation of p65 and mitogen activated protein kinase pathway proteins. The results from both the clinical and *in vitro* aspects of this study revealed that the herbs *G. glabra* and *P. grandiflorus* may offer beneficial outcomes as alternative treatments for VFNs after precise diagnosis.

## Introduction

Vocal fold nodules (VFNs) as benign vocal fold lesions are quite a challenge for otolaryngologists. Hoarse and raspy voices are distinguished symptoms for VFNs. It is popular among professional voice users like teachers or sales. The definition of VFNs are mucosal lesions occurring on both sides of the vocal fold on the border of the anterior and middle third of the vocal fold 1, 2. Such lesions are often found on the superficial layer of the lamina propia 3. Long term exposure to irritants may cause chronic laryngeal inflammation, which may further lead to the formation of VFNs.

Medical treatments, voice therapy and excision through microlaryngeal surgery are considered as VFNs treatment. Physiological, medical, and psychological factors should be considered during diagnosis and treatment 4, 5.

While voice therapy can alter voice production patterns to minimize phonotrauma, thus considered as the first-line treatment for VFNs because it typically resolves voice problems and prevents recurrence in most patients, vocal hygiene education were also proved to be a cost-effective conservative treatment especially in the countries with few speech pathologists 4,6. Both of above treatments involve education on vocal fold mechanics and etiological factors, as well as specific modifications to behaviors that exacerbate inappropriate voice production.

The compliance of the voice therapy and vocal hygiene education may be affected by various factors. For example, for the patients who live in the rural areas, the compliance and self-awareness of vocal hygiene may be hard to achieve. When the above conservative treatments failed, surgery is still the standard VFNs treatment. However, serious complications associated with surgery may occur, including damage to the vocal folds 7. Intracordal steroid injection can be beneficial for managing VFNs. However, the technique of injection on vocal folds requires a well-trained laryngologist. Nevertheless, VFNs may persist after injection.

Indeed, there are increasing patients inclined to choose complementary medicine other than original management due to concerning about the risks of surgery or side effects of steroid 8, 9. As for physiotherapy, such as massage, spinal manipulative therapy and acupuncture, there are several trials revealing that these treatments might be effective for voice disorders 10. In an small-scaled acupuncture study, anti-inflammatory significantly increased was found in the experiment group. This may be the mechanism why complementary medicine is effective to the voice disorders 11.

When it comes to traditional Chinese medicine (TCM), there are limited evidence of effects on vocal nodules and vocal polyp management. Moreover, the methodological quality remained unsatisfactory and the exact contents and the dosage were uncertain in these researches 12, 13. Therefore, it is a challenge to verify the exact effect of TCM, especially cellular mechanism.

For *in vitro* part, some herbal aqueous and ethanol extracts such as clove and ginger exhibit anti-inflammatory ability in human tonsil epithelial cells 12. There are several cell types in the upper airway. The vocal fold comprised stratified squamous epithelial cells 13. Since it is difficult to obtain healthy human vocal fold epithelial cells without risking vocal function, the fresh porcine vocal fold tissue is readily and easily available 14. Moreover, compared to other animals, the morphology of porcine vocal fold epithelium is the most similar to that of humans 15.

So far, few researches reported the effects of herbs on both vocal fold nodule and inflammatory vocal fold cells. In our study, we retrospectively evaluated the clinical effects by the clinical results of our TCM herbal treatment. Also, we further investigated the effects of these herbs in an *in vitro* study.

## Materials and Methods

### Subjects

We reviewed the medical records of 22 patients with diagnosis of VFNs which are based on videostroboscopy or pathological result from July 2018 to August 2020. Other benign vocal fold lesions were excluded from the present study, such as vocal polyps, vocal cysts, and Reinke's edema. The patients were examined with laryngeal videostroboscopy using a Kay Elemetrics Stroboscopy Unit (Model, Lincoln Park, NJ, USA) at their initial visit and were followed up 3 months after TCM treatment. The TCM treatment was given with finished herbal products (FHPs) after patient evaluation respectively. The TCM treatment was under national healthcare insurance.

Speech pathologist skilled in voice training and an otolaryngologist evaluated the recorded data. Both of them were blinded to the TCM treatment protocols. The present study was approved by the Institutional Review Board of Chiayi Chang Gung Memorial Hospital (No. 201901034B0). All participants gave written informed consent prior to the beginning of the study.

### Objective and Subjective Voice Analysis

The acoustic parameters recorded automatically were as follows: average fundamental frequency (F0, in Hertz), jitter, shimmer, and noise-to-harmonic ratio (NHR). Jitter is defined as the cycle-to-cycle variation in fundamental frequency 16, 17, and shimmer is expressed in decibels, representing the variability in the peak-to-peak amplitude. NHR is used for evaluation of dysphonic voice by measuring the amount of additive noise in the voice signal 18. The acoustic parameters were measured by the Computerized Speech Laboratory (core model CSL 4500, KayPentax, Lincoln Park, NJ, USA). The duration parameter measured in this study was the maximum phonation time (MPT). A speaker's MPT is measured by a stopwatch, as their best attempt to sustain the vowel /a/ sound at a comfortable intensity 19. Twenty-two patients completed a pre- and post-test Chinese Voice Handicap Index-10 questionnaire (VHI-C10) 20, 21. Pre- and post-TCM perceptual evaluation was performed by using the GRBAS scoring system (G = grade, R = roughness, B = breathiness, A = asthenia, and S = strain; 0 = normal, 1 = mild, 2 = moderate, and 3 = severe). It the difference between the two GRBAS scores exceeded two points, a reassessment is required. The voice parameters mentioned above were measured by a speech pathologist and an otolaryngologist in a double-blinded manner.

### Aqueous Extracts of TCM Herb Glycyrrhiza glabra and Platycodon grandifloras

The raw herbs used for the aqueous extracts (AE) were obtained from Chiayi Chang Gung Memorial Hospital (Taiwan), 20 g *Glycyrrhiza. glabra* from Batch No. 42050309 and 20 g *Platycodon. grandiflorus* from Batch No. N15529. The raw herb materials were separately macerated in 1000 mL reverse osmosis water and then boiled for 10 minutes. These filtrates were combined and concentrated under reduced pressure at 40°C by using a vacuum rotary evaporator to obtain crude AEs. Approximately 4 g of the resulting *G. glabra* AE (20% yield) and 11 g of the resulting *P. grandifloras* AE (55% yield) were stored at -20 °C before use.

### Primary Culture PVFE Cells

The porcine vocal fold epithelial (PVFE) cells for the primary culture were purchased from GeneDirex, Inc. The PVFE cells were isolated and cultured as reported 14. Epithelial cells were derived from porcine vocal folds, and then further expanded in culture. Epithelial cells identification and characterization were done by immunostaining with pan-Cytokeratin antibodies.

### MTT Assay

PVFE cells were treated with various concentrations of *G. glabra* and *P. grandiflorus*; subsequently. The percentages of metabolically active cells were determined based on the mitochondrial conversion of 3-(4,5-Dimethylthiazol-2-yl)-2,5-diphenyltetrazolium bromide (MTT) into formazine. In brief, after cells were treated with *G. glabra* and *P. grandiflorus* for various incubation times, culture media were replaced with DMEM/F-12 (1:3 ratio) containing 0.02% MTT (Sigma-Aldrich) and incubated for 4 hours; subsequently, the medium was replaced with 200 μL of dimethyl sulfoxide per well. The results were assessed in a 96-well format plate reader by measuring the absorbance at a wavelength of 595 nm on a Victor Nivo Multimode microplate reader (PerkinElmer, Akron, OH, USA).

### Inflammatory Response Induction and Culture in TCM Aqueous Extract

The PVFE cells were seeded at a density of 35×10^3^ cells/well in PLL-coated 24-well plates and incubated overnight at 35 °C for 20 hours. PVFE cells were then exposed to the inflammatory cytokine tumor necrosis factor alpha (TNF-α) 50 ng/mL for 24 hours incubation at 35 °C to trigger inflammatory reaction. In addition, the corticosteroid dexamethasone was included as a positive control anti-inflammatory drug.

### Western Blot Analysis

Cells were collected in lysis buffer containing protease and phosphatase inhibitors for protein isolation. We prepared cellular extracts by sonication. And total protein concentrations were determined for the Western blot analyses. Proteins were separated on 4%-20% Tris-Glycine gels (Invitrogen, Carlsbad, CA, USA) and transferred to nitrocellulose membranes. After the membranes were blocked in TBST (10 mM Tris-HCl buffer, pH 8.0; 150 mM NaCl; and 0.1% Tween 20) and 5% (w/v) BSA at room temperature for 60 mins, they were incubated overnight at 4 °C with antigen-specific primary antibodies. The primary antibodies used were against phospho-NF-κB p65 (Ser536) (Cell Signaling Technology, Danvers, MA, USA). The blots were under incubation with species-specific HRP-conjugated secondary antibodies for 2 hours at room temperature. And then, the proteins were visualized through incubation with a chemiluminescent substrate kit (Thermo Fisher Scientific, Waltham, MA, USA).

### Statistics

Experiments were performed at least three times. The clinical and *in vitro* data were analyzed using SPSS 13.0 (Chicago, IL, USA). The results are expressed as means ± standard deviations. *P* value less than 0.05 was considered statistically significant.

## Results

### Laryngeal videostroboscopy image outcomes

**Figure 1:**
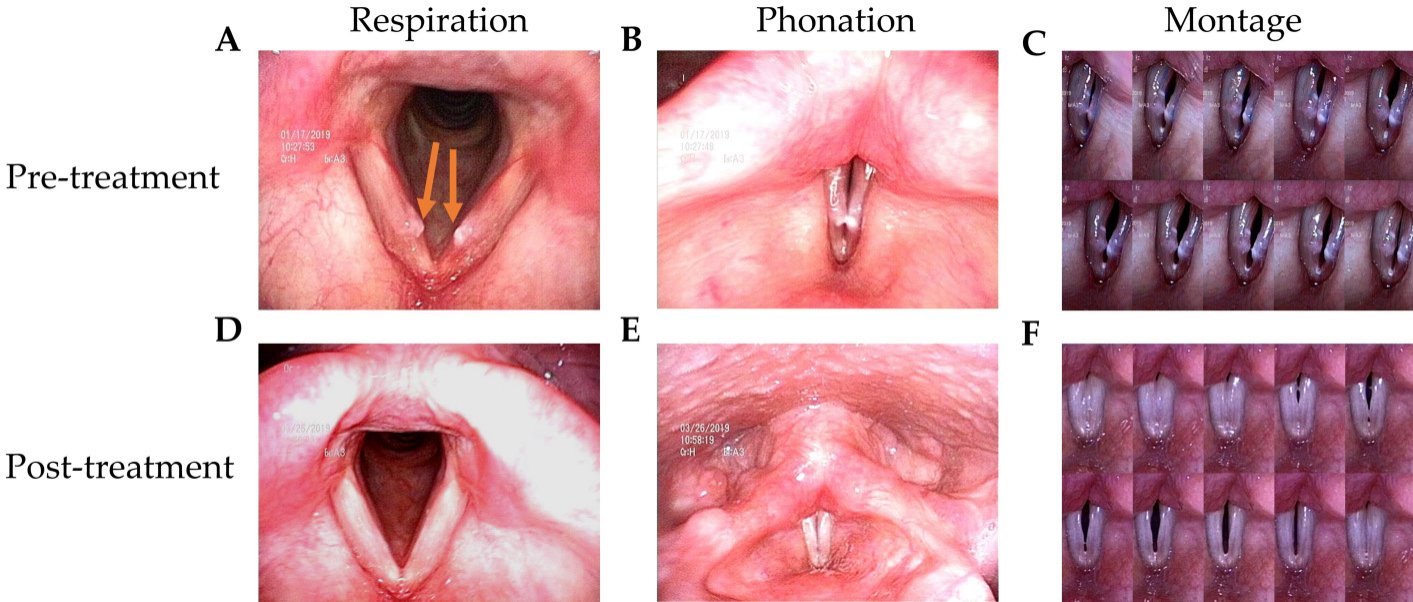
** Videostroboscopy record of a 42-year-old female patient before and after 2-week TCM treatment. (A)** Bilateral vocal nodule before TCM (respiration). **(B)** Glottal gap (phonation). **(C)** Persistent glottal gap in montage photograph during phonation. **(D)** No vocal nodule after TCM (respiration). **(E)** No glottal gap (phonation). **(F)** Montage photograph of normal glottal gap in during phonation.

The patients in this study had husky voices for more than 6 months. VFNs with mild to moderate phonatory gaps were seen under videostroboscopy. Figure 1 presents the bilateral vocal nodules at rest (Figure 1A) and the glottal gap during phonation (Figure 1B).

Patients were followed up 3 months after TCM treatment. Videostroboscopy, acoustic analysis, and perceptual assessment were conducted. Figure 1D and 1E presents the post-TCM glottal gap at rest and during phonation, respectively. We found that after TCM treatment, most patients achieved marked improvements in glottal closure, mucosal wave, and the amplitude of mucosal wave under videostroboscopy. No additional morbidities or complications were observed during treatment.

### Objective and Subjective Voice Parameters

The acoustic analyses before and after TCM herb treatments are presented in Table 1. No significant differences were noted between MPT and NHR among the two groups (*P* > 0.05). Subjective VHI-C10 questionnaire scores, assessed by a blinded voice therapist, decreased significantly after TCM treatment. Jitter, shimmer, and GRBAS scores also improved significantly. A significant decrease was also observed in pre- and post-TCM perceptual assessments, which were conducted according to total GRBAS scores (Table 2).

### TCM Medication Review

**Figure 2:**
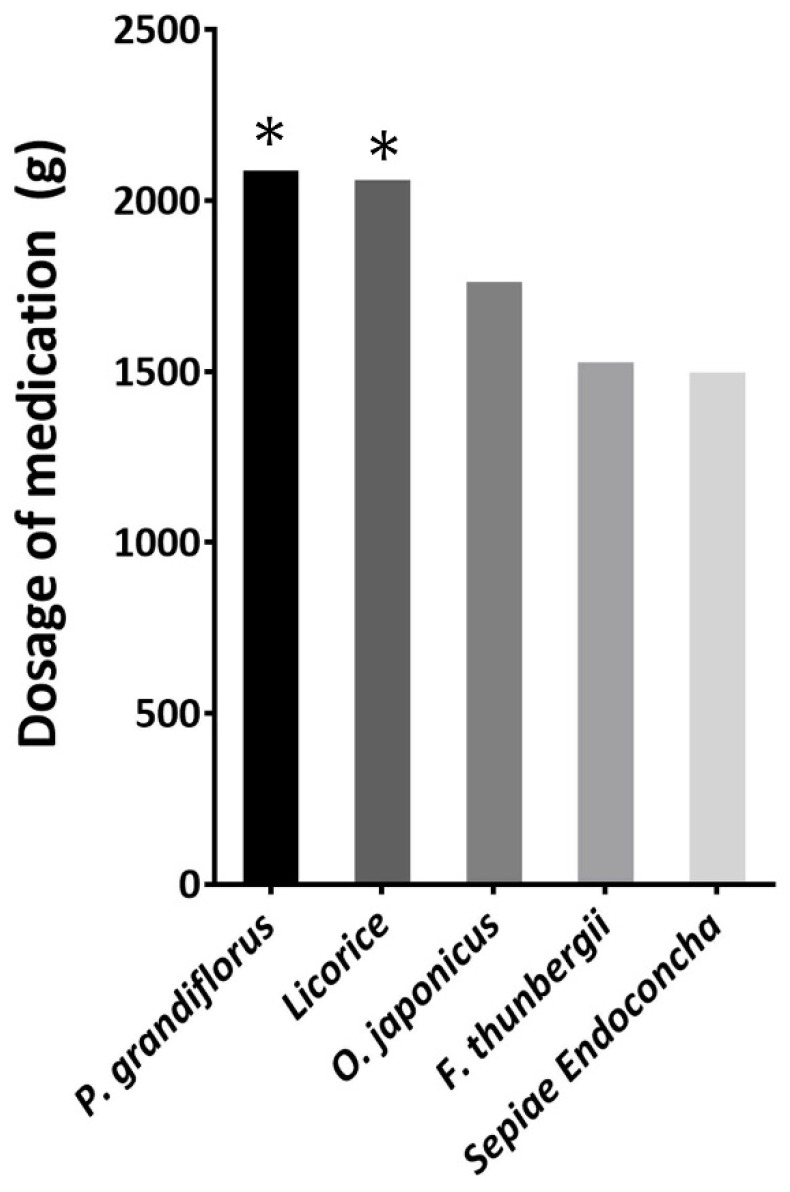
** Total dosage ranking of the medication prescribed to 22 patients.** The total prescribed amount of *P. grandifloras* and licorice are more than 2000 grams.

We pooled the prescribed medications of the study participants and calculated the total doses. The following herbs were prescribed with total dosages of >1500 g (Figure 2): *Platycodon. grandiflorus*, *Glycyrrhiza glabra*(licorice), *Ophiopogon japonicus*, *Fritillaria thunbergii*, and Sepiae Endoconcha (cuttlebone). The total prescribed amount of *P. grandifloras* and licorice are more than 2000 grams. In our prescription pattern, a 1:1 ratio of *G. glabra* to *P. grandiflorus* may be more beneficial for VFNs patients. The average duration and doses of *P. grandifloras* and licorice for the patients are summarized in Table 2.

### P65 and mitogen-activated protein kinase Pathway Inhibition by Glycyrrhiza glabra and Platycodon grandiflorus *in vitro*

**Figure 3:**
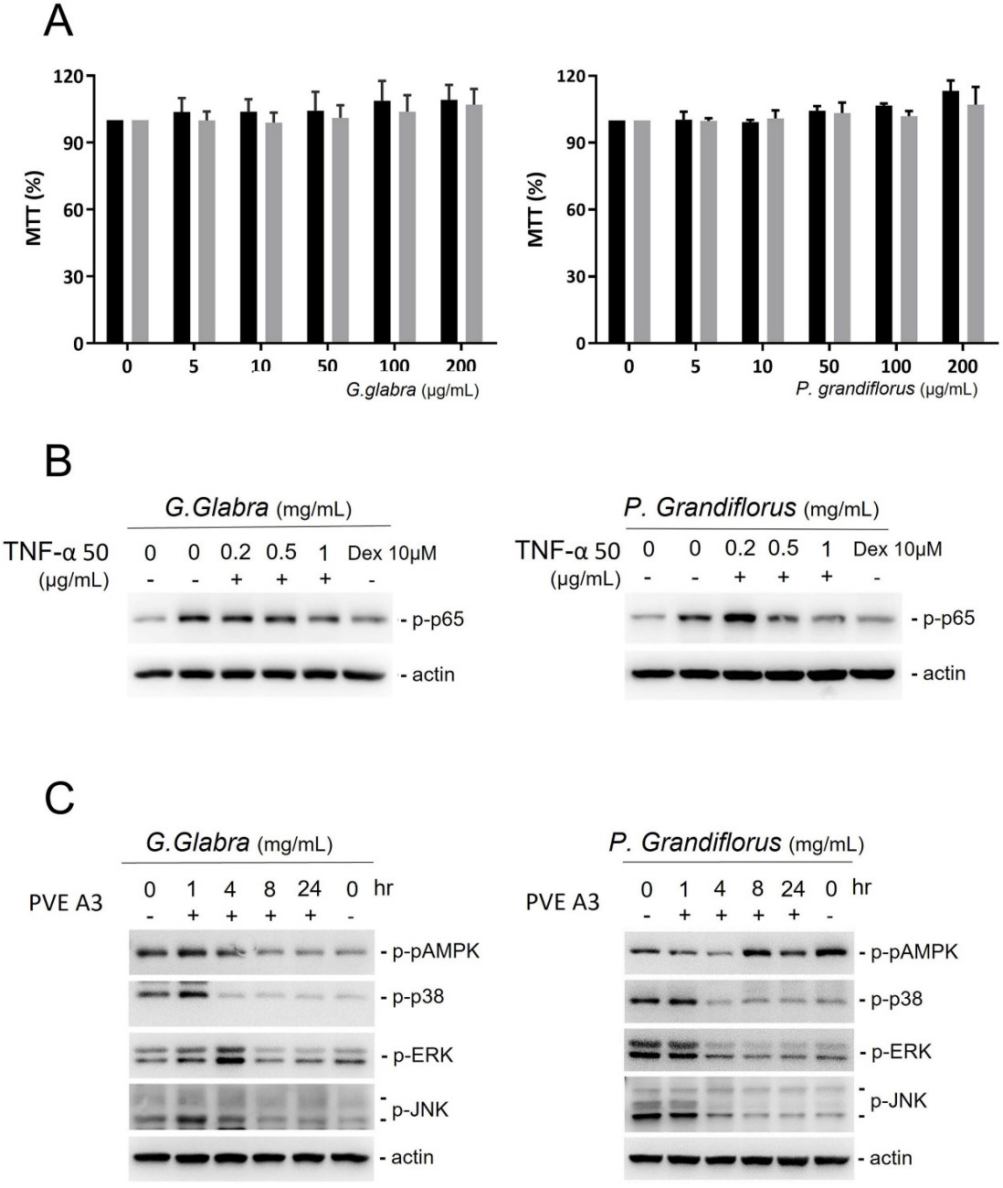
** PVFE cells were treated with *G. Radix* and *P. grandiflorus*. (A)** *G. Radix* and *P. grandiflorus* did not result in cell toxicity in PVFE cells. **(B)** *G. Radix* and *P. grandiflorus* reduced the expression of phosphorylated p65 in TNFα-treated cells. **(C)** P38, ERK, and JNK pathways were inhibited by* G. Radix* and *P. grandiflorus*, but their effect on the AMPK pathway was unclear.

*G. glabra* and *P. grandiflorus* did not hinder the growth of PVFE cells even at high concentrations (Figure 3A). In PVFE cells treated with 50 ng/mL TNFα, NF-kβ p65, a downstream target of the mitogen-activated protein kinase (MAPK) pathways, was stimulated by phosphorylation. *G. glabra*, *P. grandiflorus* and the corticosteroid dexamethasone could reduce the expression of phosphorylated p65 in TNFα-treated cells (Figure 3B). To further study the mechanism of the anti-inflammatory effect of TCM, the change in p-AMPK, p38, extracellular signal-regulated kinases (ERK), and Jun N-terminal kinases (JNK) in response to TCM treatment in VFE cells was investigated (Figure 3C). *G. glabra* and *P. grandiflorus* significantly inhibited the p38, ERK, and JNK pathways, but their effect on the MAPK pathway was unclear.

## Discussion

In this study, we retrospectively collected medical records of 22 vocal fold nodule patients treating with TCM from July 2018 to August 2020. We found the higher prescription frequency and greater consumed weight of *P. grandiflorus* and licorice.

For the clinical part, in the TCM outpatient clinics, the patients with vocal nodules were referred from the otolaryngologists for TCM medication prescription. The composition varied for every single patient since TCM medication was generally thought to be more individualized.

The 22 patients regularly returned to TCM clinic every 2 to 4 weeks. In the meanwhile, they were required to come back for voice analysis. The medication we prescribed was in the form of granulated compounds called finished herbal products (FHP).

The finished herbal products (FHP) we prescribed are granulated compounds concentrated from Chinese herbal remedies, including single herbs and herbal formulae 22. They are officially approved in the national healthcare systems not only in Taiwan, but also in Japan, Korea and Mainland China. They are widely used in the East Asia 23. Compared to traditional herbal decoction, the form of FHP is more portable for the patients and easier for us to calculate the weight consumed by our patients.

Since the medication should be different from patient to patient, we found that the prescription for these patients had similarity in their components. Therefore, after chart review for the TCM medication, we can see from Figure 2, the most used herbs were *G. glabra* and *P. grandifloras*. The total amount was more than 2000 grams.

*G. glabra*, the licorice specie we used here, was prepared by Chiayi Chang Gung Memorial Hospital (Taiwan). *G. glabra* is grown in Eurasia, northern Africa and western Asia 24. Its rhizomes and roots are the most important medicinal parts, which have been reported to be used alone or in combination with other herbs for treating multiple diseases such as digestive disorders, respiratory tract disorders, epilepsy, fever, sexual disability, and etc 25. *P. grandiflorus* contains numerous amino acids, vitamins, and trace elements. Also, it has the ability to treat the following disorders: cough and asthma relief, antitumor activity, anti-inflammatory and antibacterial effects, antioxidation, liver protection, and immunity enhancement 26. Both *G. glabra* and *P. grandiflorus* can be used as food and flavoring agents.

The combined use of these two herbs can be traced to ancient China. In the Chinese classic *Shang Han Lun* (English title: Treatise on Cold Damage Diseases, written by Zhang Zhong-Jing, 150-219 A.D.), the two herbs were combined in a 2:1 ratio and were used mainly to treat sore throat and Fei Yon (pulmonary abscess) 27, 28. Pharmacologically, they can affect the metabolic process of each other, to improve the bioavailability of their compounds 29, 30.

In second part of the study, we used these two herbs for porcine PVFE culture. And the two herbs significantly inhibited the P38, ERK, and JNK pathways in TNF-α inflammatory process induced by TNF-α. For the design of our experiments, since inflammation is commonly observed in patients with chronic voice disorders, such as vocal fold nodules. It was demonstrated that an immediate increase of inflammatory cytokines, including TNF-α, is observed in response to vocal fold injury. The effect of TNF-α on the vocal fold epithelium upregulates mucin expression 14. TNF-α may activate the NF-κB p65 pathway in PVFE cells, but this inflammatory pathway is inhibited by the corticosteroid dexamethasone (Dex) and high doses of *G. glabra* and *P. grandiflorus*. This means that the anti-inflammatory effect of *G. glabra* and *P. grandiflorus*. on the vocal folds depends on their effect in vocal epithelium cells.

MAPK families are critical in complex cellular programs like proliferation, differentiation, development, transformation, and apoptosis. The MAPK pathways involve a series of protein kinase cascades, which play a crucial role in the regulation of cell proliferation. The three major MAPK families, namely mammalian ERKs, c-JNK, and p38 kinases, are activated by many stimuli. JNK and p38 are activated by cytokines and stressors, and ERK is mainly activated by growth factors 31. In our study, it revealed that *G. glabra* and *P. grandiflorus* downregulated the activation of p38, p-ERK, and p-JNK. This result explained the effectiveness of *G. glabra* and *P. grandiflorus* on vocal nodules by downregulation of the MAPK pathway 32.

However, there were some limitations in this study. Our sample size is relatively small. Also, the dosage and duration varied, which may be related to individual difference. For *in vitro* study, though the above inflammatory pathway down-regulation was seen, whether there exists synergistic effect or other interaction between the herbs are still unknown in clinical practice. Further prospective and functional studies are needed to confirm the results of this study and clarify the mechanism of action.

In conclusion, by reviewing clinical patient data and conducting an *in vitro* study, we confirmed that TCM herbal treatment is an alternative treatment option for VFNs. Patients' voices exhibited improvement after daily TCM herb use, and VFNs were reduced. From the above results, we may imply that our most prescribed herbs *G. glabra* and *P. grandiflorus* as potential VFNs alternative treatment.

## Figures and Tables

**Table 1:** Demographic data and pre- and post-TCM acoustic analysis results of study patients

Characteristics	Total patients (n = 22)		
	Pre	Post	*p* value
Sex (Male/Female)	4/18		
Mean age (Min-Max)	56 (32-75)		
Mean follow up time (days)	77 (12-150)		
MPT (sec)	9.02 ± 4.27	8.75 ± 4.52	0.770
Jitter (%)	2.86 ± 1.77	1.80 ± 1.28	0.001*
Shimmer (dB)	0.67 ± 0.48	0.53 ± 0.50	0.003*
NHR	0.19 ± 0.10	0.16 ± 0.10	0.017*
VHI-C10	17.6 ± 8.06	6.6 ± 6.33	<0.001*
GRBAS	5.91 ± 3.73	2.93 ± 3.34	<0.001*
G	1.39 ± 0.74	0.84 ± 0.82	0.005
R	1.39 ± 0.74	0.75 ± 0.74	0.002
B	1.14 ± 0.77	0.57 ± 0.73	0.002
A	0.95 ± 0.84	0.36 ± 0.66	0.001
S	1.05 ± 0.84	0.41 ± 0.67	<0.001

**Table 2:** The duration and dosage for each patient of Licorice and *P. grandiflorus* obtained from a medication chart review of 22 patients

	Licorice	*P. grandiflorus*
Duration (days)	63.95 ± 32.44	62.73 ± 32.89
Median (range)	69.5 (14-143)	63 (14-143)
Dosage (grams)	97.09 ± 78.45	93.98 ± 79.73
Median (range)	68.5 (5.61-296)	60 (7.14-296)

## Abbreviations

VFNs: Vocal fold nodules
VHI-C10: Chinese Voice Handicap Index-10
MDVP: multidimensional voice program
PVFE: porcine vocal fold epithelial
*G. glabra* (licorice): Glycyrrhiza glabra
*P. grandifloras*: Platycodon grandifloras
TCM: traditional Chinese medicine
F0: fundamental frequency
NHR: noise-to-harmonic ratio NHR
MTT: 3-(4,5-Dimethylthiazol-2-yl)-2,5-diphenyltetrazolium bromide
MAPK: mitogen-activated protein kinase
ERK: extracellular signal-regulated kinases
JNK: Jun N-terminal kinases
FHP: finished herbal products
Dex: dexamethasone
